# Structural basis for the substrate selectivity of *Helicobacter pylori* NucT nuclease activity

**DOI:** 10.1371/journal.pone.0189049

**Published:** 2017-12-04

**Authors:** Louisa Celma, Christopher Corbinais, Julien Vercruyssen, Xavier Veaute, Inès Li de la Sierra-Gallay, Raphaël Guérois, Didier Busso, Aurélie Mathieu, Stéphanie Marsin, Sophie Quevillon-Cheruel, J. Pablo Radicella

**Affiliations:** 1 Institute for Integrative Biology of the Cell (I2BC), CEA, CNRS, Université Paris-Sud, Gif-sur-Yvette cedex, France; 2 Institute of Molecular and Cellular Radiobiology, CEA, Fontenay aux Roses, France; 3 UMR967 INSERM/CEA/Universités Paris Diderot et Paris-Sud, Fontenay aux Roses, France; Universidad de Santiago de Compostela, SPAIN

## Abstract

The Phospholipase D (PLD) superfamily of proteins includes a group of enzymes with nuclease activity on various nucleic acid substrates. Here, with the aim of better understanding the substrate specificity determinants in this subfamily, we have characterised the enzymatic activity and the crystal structure of NucT, a nuclease implicated in *Helicobacter pylori* purine salvage and natural transformation and compared them to those of its bacterial and mammalian homologues. NucT exhibits an endonuclease activity with a strong preference for single stranded nucleic acids substrates. We identified histidine124 as essential for the catalytic activity of the protein. Comparison of the NucT crystal structure at 1.58 Å resolution reported here with those of other members of the sub-family suggests that the specificity of NucT for single-stranded nucleic acids is provided by the width of a positively charged groove giving access to the catalytic site.

## Introduction

The Phospholipase D (PLD) superfamily is ubiquitous in most forms of animal, plant and bacterial life. It is composed of a very large set of enzymes involved in numerous biochemical pathways like signal transduction, mitosis, metabolism and secretion [1–3]. The members of this superfamily support various biochemical functions as phospholipases, cardiolipin synthases, phosphatidylserine synthases, nucleases, toxins and virus envelope proteins. The family is defined by the “HxK(x)_4_D(x)_6_GSxN” sequence signature where the histidine is proposed to act in all PLD enzymes as a nucleophile in a phosphodiester bond cleavage. The proposed chemical mechanism of the phosphoryl transfer includes the formation of a five-coordinate phospho-histidine intermediate at the active site [4].

The PLD proteins are organized as symmetrical dimers and are characterized by the canonical α/β PLD fold. They can be classified into two major sub-families: the first class is represented by PLDs that hydrolyse the terminal phosphodiester bond of phospholipids to phosphatidic acid and a hydrophilic constituent. This PLD subfamily also includes proteins that catalyse a transphosphatidylation reaction in the presence of phosphatidylcholine and a short-chained primary or secondary alcohol [5]. The second class is composed of endonucleases like Zucchini, Nuc or Bfil which act on nucleic acids [6].

From the structural point of view, the first PLD sub-family members accommodate the lipid substrate in a deep hydrophobic pocket covered by two flexible loops limiting the access to the internal catalytic pocket to a small round area [7]. The PLDs from the endonuclease sub-family possess a positively charged and elongated groove that is well adapted for the interaction with negatively charged nucleic acids. The width of this groove varies with the nature of the cognate substrate: the non-specific nuclease Nuc harbours a large groove that can accommodate single stranded as well as double stranded substrates [1], whereas Zucchini (Zuc), which is specific for RNA, has a narrower groove [7–9].

**Fig 1:**
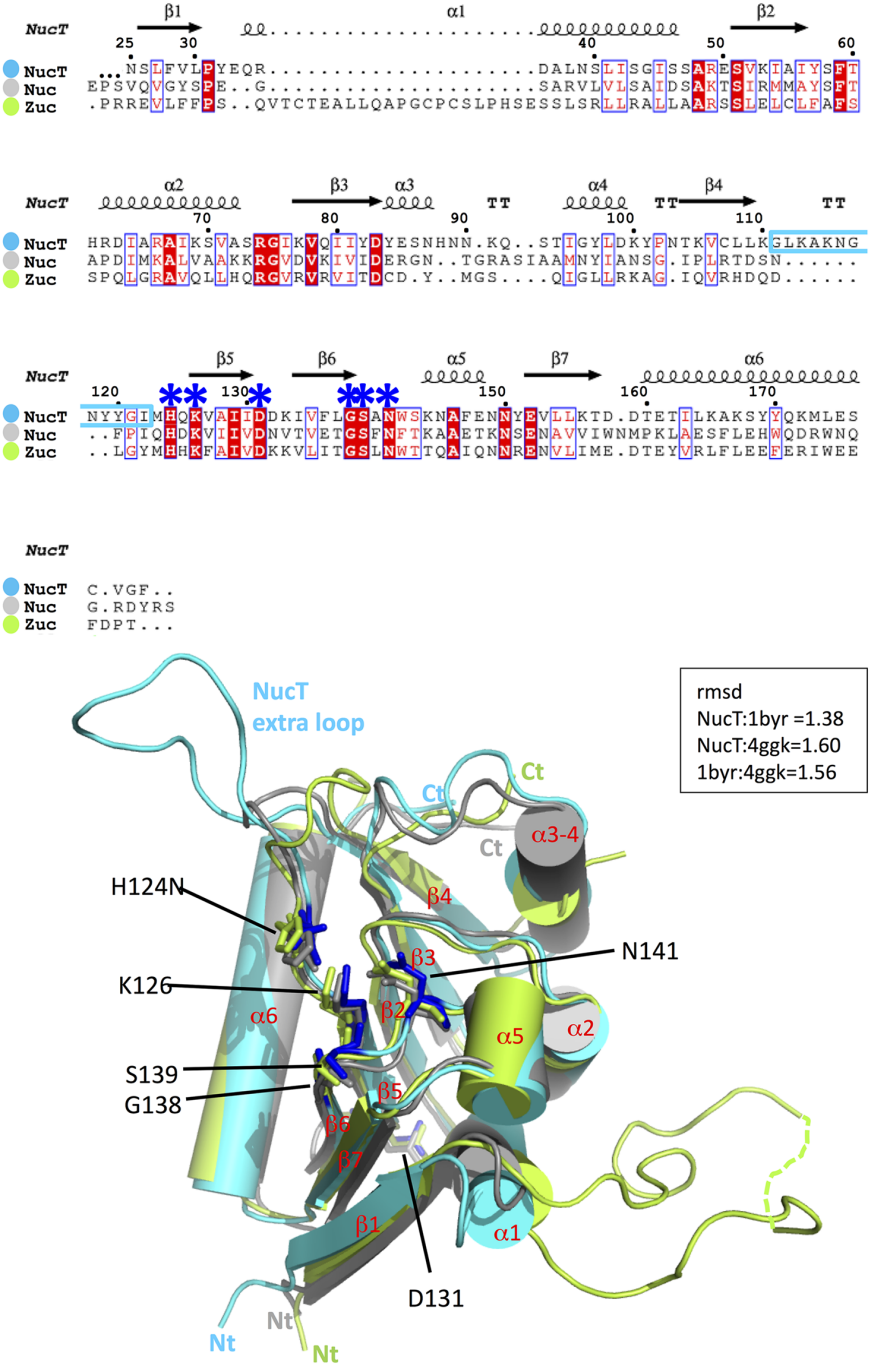
X-ray structure of the NucT monomer. **(A)** Structure-based sequence alignment of NucT [25-end], Nuc [24-end] and Zucchini [36–209]. The multialignment was generated by Clustalw2 [32]. The figure was generated using ESPRIPT [33]. The secondary structure elements of NucT are in black on the top of the multialignment. The functional residues are pointed by blue stars. The sequence of the extra loop of NucT is boxed in cyan. **(B)** Structure superimposition of NucT^H124N^ (cyan) with Nuc (PDB ID 1byr, grey) and Zucchini (PDB ID 4ggk, green). The amino acids involved in the active site are in sticks. The extra loop of NucT is indicated.

Hp0323 (NucT) encodes a nuclease from *Helicobacter pylori* belonging to the endonuclease PLD sub-family (Fig 1). So far, NucT has been implicated both in the process of natural transformation and in the purine salvage pathway. The presence of NucT in the periplasm as a membrane-associated protein together with the effect of its inactivation, albeit modest, on transformation frequencies, suggested a role for this nuclease in *H*. *pylori* competence [10]. However, NucT has no homology with EndA, the ββα-metal finger motif nuclease responsible for the DNA processing during the transformation process in other bacterial species (active center signature DRGH) [11]. Interestingly, while *Wolinella succinogenes* has an orthologue of NucT (S1 Fig), *Campilobacter jejuni*, a species closely related to *Helicobacter* and also naturally transformable, does not present one. *H*. *pylori* lacks several genes involved in the *de novo* synthesis of purine nucleotides [12] and must consequently salvage purines from the human gastric epithelium in order to grow [13]. NucT has been proposed to be involved in the initial steps of purine recycling [14].

Here, we compared the nuclease activity and the high resolution crystal structure of NucT to those of Nuc and Zucchini, also members of the PLD nucleases sub-family. Our results suggest an explanation for the preference of NucT for single-stranded substrates and for its high affinity for DNA.

## Materials and methods

### Cloning and mutagenesis

The full-length NucT encoding sequence was amplified by PCR using *Helicobacter pylori* 26695 purified genomic DNA as a template. The resulting PCR product was sub-cloned in pMal-p2X plasmid (New England Biolabs) digested by *Xmn*I-*Xba*I. To delete the signal peptide spanning the 23 first amino-acids of NucT protein, the previous plasmid was amplified by PCR with overlapping primers (forward primer: 5’–AAAAACAGCTTATTTGTCTTACCTTATG– 3’ and reverse primer: 5’–GTAAGACAAATAAGCTGTTTTTTGAAATCCTTCCCTCGATC– 3’) generating pMal-p2X-NucT-24-180. The initial Factor Xa recognition site encoding sequence of pMal-p2X-NucT-24-180 was replaced by a TEV protease recognition site encoding sequence and a linker by PCR with overlapping primers (forward primer: 5’–GAATTCAAAAACAGCTTATTTGTCTTACCTTATG– 3’ and reverse primer: 5’–CAAATAAGCTGTTTTTGAATTCTGAAATGCCCTGAAAATACAGGTTTTCCCCGAGGTTGTTGTTATTG– 3’) generating the pMal-p2X-TEV-EF-NucT-24-180 plasmid. Finally, site directed mutagenesis of the His124 residue in Asn was conducted by PCR with overlapping primers (forward primer: 5’–AACCAAAAAGTAGCGATCATTG– 3’ and reverse primer: 5’–GATCGCTACTTTTTGGTTCATGATGCCGTAATAATTCC– 3’). All PCR were performed with Phusion DNA polymerase (NEB).

### Protein over-expression and purification

Twenty-five milliliters of an overnight culture of *E*. *coli* K12 TB1 (NEB) transformed by either by pMAL-p2X-TEV-EF-NucT-24-180 or pMal-p2X-TEV-EF-NucT-24-180-H124N plasmid were used to inoculate two liters of LBx2 broth (Lennox) supplemented by 1% glucose and ampicillin (100 μg/ml). After growth at 37°C, cells were harvested at A600 ~1, centrifuged and suspended in LBx2 broth + ampicillin pre-warmed at 37°C. For recombinant protein expression, IPTG was added to a final concentration of 1 mM and cells were grown for additional 3h at 37°C. Upon harvesting by centrifugation at 5000 x g for 15 minutes, cell pellets were suspended in 640 ml of 30 mM Tris-HCl pH 8.0, 20% sucrose. EDTA was added to a final concentration of 1 mM and the cells were incubated for 10 minutes at 30°C under shaking (240 rpm). After centrifugation at 8000 x g at 4°C for 15 min, the pellet was suspended in 80 ml of ice-cold 5 mM MgSO_4_ and incubated for 10 min at 4°C under shaking (245 rpm). After centrifugation at 8000 x g at 4°C, the supernatant corresponding to the cold osmotic shock fluid was recovered, was supplemented with 20 mM Tris HCl pH 7.4 and then loaded onto an amylose resin (NEB). After extensive washing with buffer A (20 mM Tris HCl pH 7.4 at 20°C; 200 mM NaCl, 1mM EDTA; 1 mM DTT), the fusion protein was eluted in buffer A supplemented with 10 mM maltose. After cleavage with TEV protease, NucT was separated from the MBP and TEV on a Resource S using a NaCl gradient from 100 to 1000 mM, in buffer B (50 mM Tris-HCl pH 8.0 at 4°C; 1 mM DTT). NucT was eluted at about 200 mM NaCl (S2A Fig).

### Crystallisation, data collection, model building and refinement

The crystals of HpNucT^H124N^ were obtained in a 1:1 ratio mixture of 17 mg/ml protein solution, in a buffer composed of 200 mM NaCl, 50 mM Tris-HCl pH 8.0, 1 mM DTT and crystallisation liquor containing polyethylene glycol 4,000 28% (w/v), 0.2 M ammonium sulphate and 0.1 M sodium acetate pH 6.0, at 18°C. Crystals appeared within 2 days. The crystals were cryo-protected by a brief soaking into the crystallisation liquor supplemented with 30% (v/v) glycerol and then flash frozen in liquid nitrogen. Data were collected at 100K on the ID23-1 beamline at ESRF synchrotron (Grenoble, France) and processed with the XDS package [15]. Data were recorded to a resolution of 1.58 Å. Crystals belonged to the P1 space group with 12 copies of NucT^H124N^ in the asymmetric unit (Table 1). The structure was determined by molecular replacement using PHASER [16] and the structure of the Zucchini endoribonuclease from mouse as a search model (PDB ID: 4GGK). Initial refinement was performed using REFMAC of the CCP4 suite [17,18]. Later rounds of refinement were alternated with cycles of manual rebuilding with COOT [19] and the final refinement was carried out with REFMAC. Validation of the structures was performed using the PDB validation server.

**Table 1:** Data collection and structure refinement statistics.

Data collection	NucT^H124N^-[24–180]
Space group	P1
Unit cell parameters	***a*** = 61.25Å ***b*** = 71.26 Å ***c*** = 110.29Å **α** = 81.38° **ß** = 74.32° **γ** = 82.37°
Redundancy†	3.5 (3.5)
Resolution range (Å)†	47.41–1.58 (1.67–1.58)
Completeness (%)†	93.0 (89.5)
R_sym_ (%)§†	6.0 (50.5)
CC1/2	99.7 (85.9)
**Refinement**	
Resolution range (Å)	47.41–1.58 (1.62–1.58)
R/R_free_(%)	20.126/24.260 (32.1/34.3)
**Geometry statistics**	
r.m.s. deviation bonds (Å)	0.021
r.m.s. deviation angles (°)	2,065
Average B-factor (Å^2^)	25,741
**Ramachandram plot**	(coot)
Most favored (%)	96
Additionally allowed (%)	4

^†^ Values in parentheses refer to the highest resolution shell (1.67–1.58Å)

^§^ *R*_sym_ = Σ_*h*_Σ_*i*_ |(*I*)_*h*_*−I*_*h*,*i*_| /Σ_*h*_Σ_*i*_ *I*_*h*,*i*_, where (*I*)_*h*_ is the mean intensity for reflection *I*_*h*_ and *I*_*h*,*i*_ is the intensity of an individual measurement of reflection *I*_*h*_.

Exploration of the 3D structures was performed using the following tools: the Dali server [20], I-TASSER [21] and the Swiss-modeling servers [22], PyMOL Molecular Graphics System [23].

### Substrates for activity tests and EMSA

**Fig 2:**
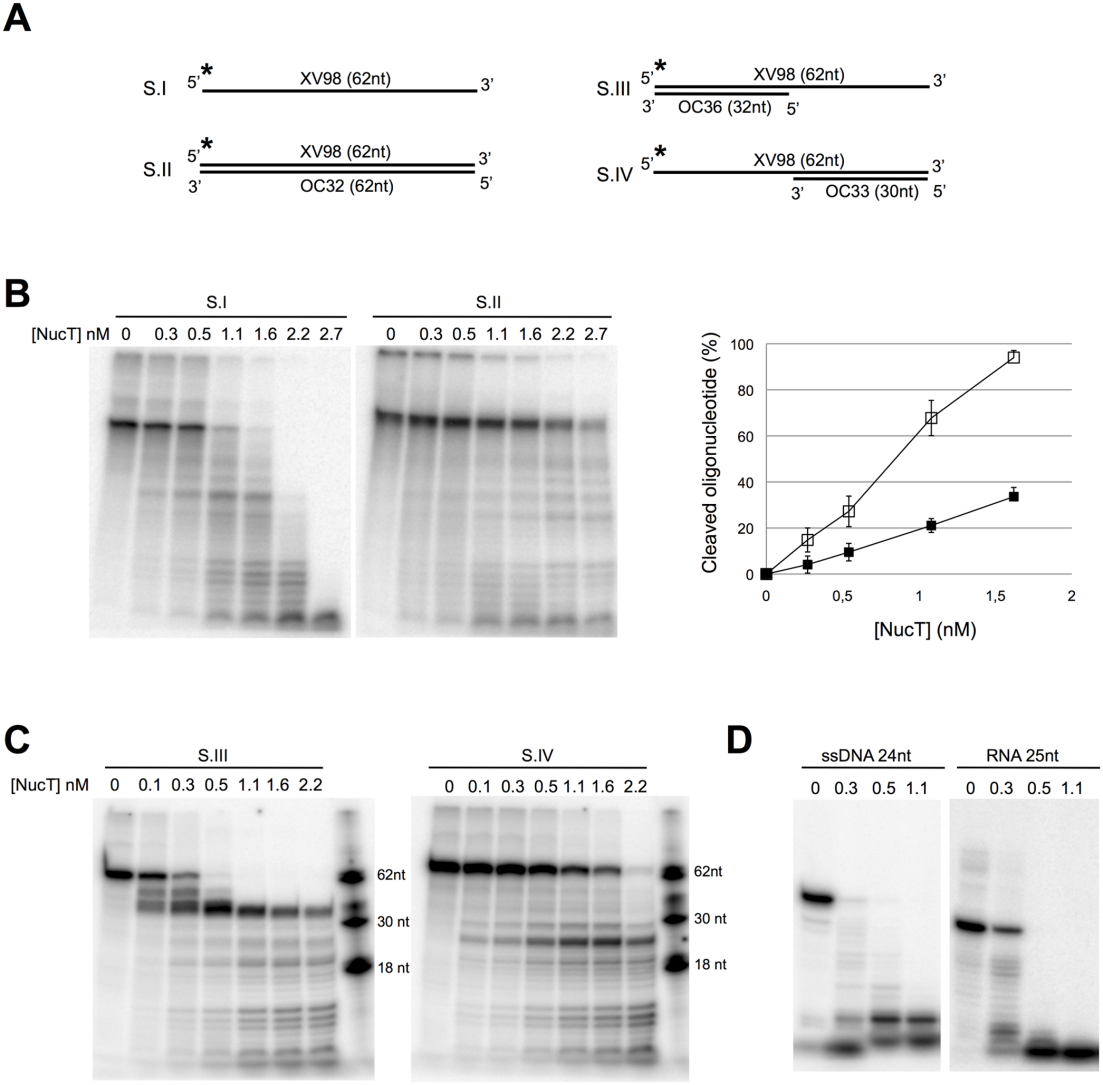
Nuclease NucT prefers single-stranded nucleic acids. **(A)** Schematic representation of the different 5’ radiolabelled DNA substrates (S.) used. See also the S1 Table. Asterisks indicate the radiolabelled 5’ end of the XV98 oligonucleotide common to the substrates used in **B** and **C**. **(B to D)** Nuclease activity tests. NucT at the indicated concentrations was incubated for 30 min at 37°C with the indicated substrate. Quantification of three independent cleavage experiments on SI (open squares) and SII (black squares) is reported as a graph in the B right panel. In **D** NucT activity was tested on the 24-mer single-stranded osf344 DNA oligonucleotide (ssDNA 24nt) or a 25-mer RNA Hxv82 (RNA 25nt), both radiolabelled in 5’. The samples were run on denaturing PAGE as described in Materials and methods.

**Fig 3:**
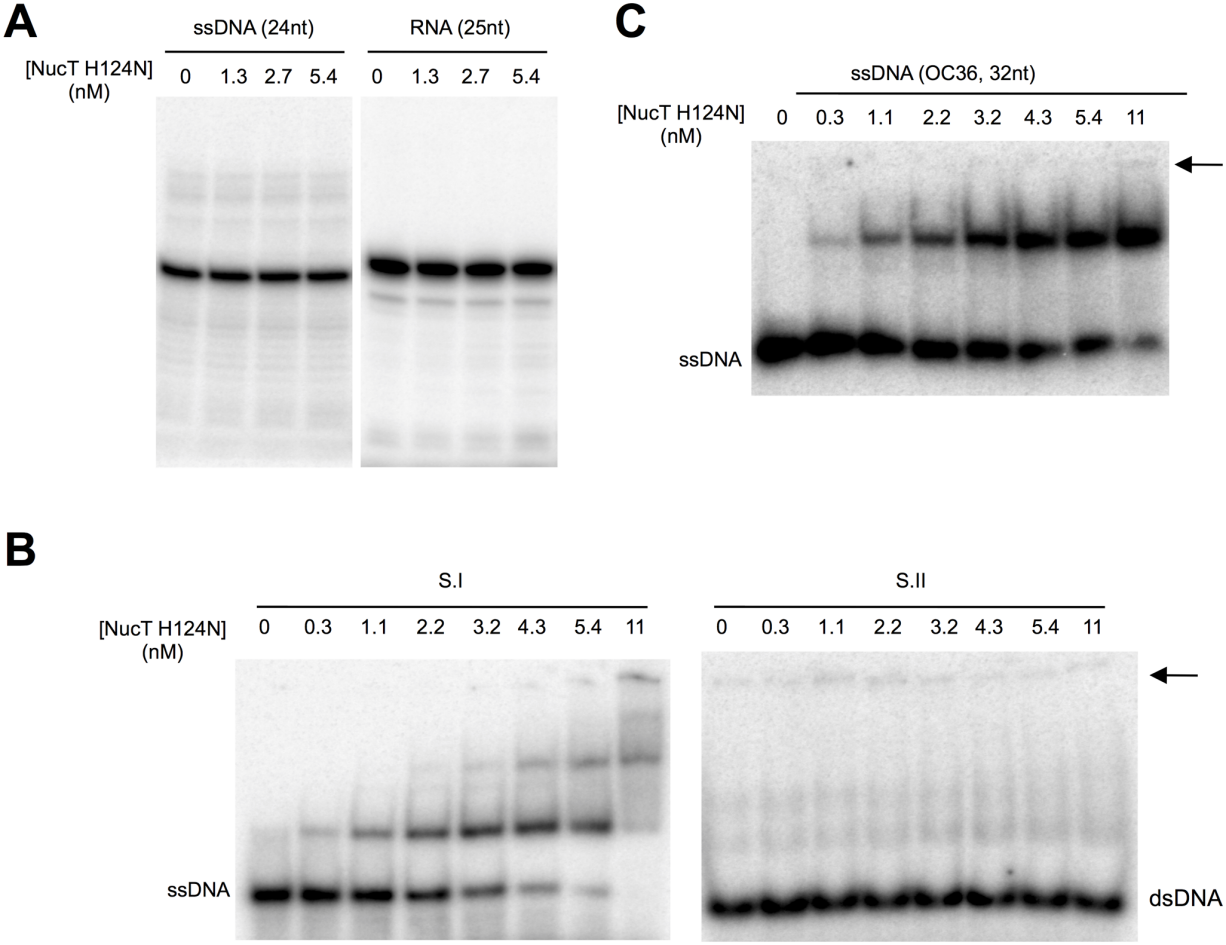
NucT catalytic site mutant. **(**A) NucT^H124N^ nuclease activity tests on ssDNA (24-mer osf344) and RNA (25-mer Hxv82). **(B** and **C)** Gel shift analysis of NucT^H124N^ binding with different DNA. Different concentrations of NucTH^124N^ were incubated for 10 min at 4°C with substrate I (B left panel) or II (B right panel) or a 32-mer single-stranded DNA oligonucleotide (C). Nucleoprotein complexes were separated on native PAGE, as described in Materials and methods. The arrow indicates the position of the wells.

Sequences of DNA and RNA oligonucleotides used are indicated in the S1 Table. Labelling at the 5’ end of oligonucleotides was performed using [γ-^32^P]-ATP and T4 polynucleotide kinase. Labelling at the 3’ end was performed using [α-^32^P]-ATP and terminal nucleotidyl-transferase. (New England Biolabs as recommended by the supplier). The different molecules used as substrates (S.I to S.VII) are represented in Figs 2A and 3A. Except for S.I and S.V, the substrates resulted from annealing of the 5’ radiolabelled XV98 oligonucleotide [24] with different oligonucleotides as indicated in Figs 2A and 3A. Unlabelled oligonucleotides were added in a 1.1-fold excess in annealing buffer A (10 mM Tris-HCl pH 8.0, 1 mM EDTA, 100 mM NaCl). The mixture was heated at 95°C for 5 min and cooled slowly to room temperature. The annealed products were tested by loading on an 8% polyacrylamide gel (30:1) and electrophoresed in 1xTBE buffer. After electrophoresis, gels were directly imaged a Typhoon system (GE healthcare).

### Nuclease activity tests

Labelled substrates (0.4 nM) were incubated with purified NucT or NucT^H124N^ protein in 10 μl of R buffer (50 mM Tris-HCl pH 6.0, 1 mM DTT, 1 mM EDTA, 150 mM NaCl, and 0.1 mg/ml BSA,). The amounts of protein used, the temperatures and the incubation times are indicated in the figures’ legends. Reactions were stopped on ice by the addition of 5 μl of loading buffer (80% deionized formamide, 50 mM Tris-borate, 1 mM EDTA, 0.1% xylenecyanol and 0.1% bromophenol blue, pH 8.3). The reaction products were separated on 20% polyacrylamide (20:1) gels containing 8 M urea. After electrophoresis, gels were directly scanned with a Typhoon system (GE healthcare).

### Electrophoretic mobility shift assay

Labelled oligonucleotides (0.4 nM) were incubated on ice for 10 min with purified NucT^H124N^ protein in 10 μl of E buffer (50 mM Tris-HCl pH 7.0, 1 mM DTT, 1 mM EDTA, 50 mM NaCl, 12.5% glycerol and 0.1 mg/ml BSA). The amounts of protein used are indicated in the figures captions. The samples were loaded on 6% polyacrylamide (29:1) gels containing 5% glycerol and 0.25xTBE and migrated in 0.25xTBE. After electrophoresis, gels were directly scanned with a Typhoon imaging system (GE healthcare). The results were quantified with ImageJ Software and the apparent *K*_*D*_ value was determined according to Riggs et al [25,26].

## Results and discussion

### The 3D fold of NucT is highly conserved from bacteria to humans

Despite a relatively low sequence homology of about 20% identity, *Helicobacter pylori* NucT sequence analysis using the HHpred server (toolkit.tuebingen.mpg.de/hhpred/) [27] predicted with very high confidence (probability scores up to 99.7%) a structural homology with Nuc, an endonuclease from *Salmonella thyphimurium*, as well as with mitochondrial-associated proteins involved in RNA processing, such as Zucchini. Nuc and Zucchini display the conserved HxK(x)_4_D(x)_6_GSxN catalytic site motif characteristic of the PLD superfamily [9], also present in NucT (residues 124 to 141, corresponding to residues 100 to 117 in our structure, Fig 1A). Mutation of the conserved histidine in both mouse and fly Zucchini resulted in catalytically inactive enzymes indicating a critical role of this residue [8,9]. This catalytic histidine corresponds to H124 (100 in our structure) in NucT.

To better understand the relationship between NucT and the other nucleases of the PLD superfamily, we solved the X-ray structure of the H124N inactive mutant deleted of the N-terminal peptide signal (see below), at a resolution of 1.58 Å. The statistics for data collection and refinement are summarized in Table 1. Twelve identical copies of NucT are present in the asymmetric unit of the crystal (rmsd ranged from 0,7413 to 0,8494 Å taken monomer A to the 11 others), forming 6 identical dimers (rmsd from 0,5647 to 0,6868 Å) with a 2-fold rotational symmetry axis. All of the members of the PLD family are described as similar dimers.

As expected from the HHpred simulations, the following structures deposited at the PDB share strong structural similarity with NucT: Nuc encoded by the plasmid pKM101 from *Salmonella typhimurium* (StNuc, PDB ID: 1byr/1bys, ds and ssDNA nuclease [1,2]), Zucchini endonuclease from *D*. *melanogaster* (DmZuc, PDB ID: 4gel/4gem and 4h4a, piRNA biogenesis [6,9]), and from mouse (mZuc, PDB ID: 4ggj/4ggk, piRNA biogenesis [8]). The root-mean-square deviation (RMSD) between NucT monomer (calculated on almost the entire protein) and these structures (monomers) are respectively of 1.38 (NucT:1byr), 1.60 (NucT:4ggk) and 1.42 (NucT: 4gel) while 1byr and 4ggk superimpose with a rmsd of 1.56 (Fig 1B). The active site of NucT and of its homologues is carried by the loops that link its seven-stranded β-sheet, itself surrounded on one side by 5 α-helices and on the other side by one long α-helix (Fig 1B). The HxK(x)_4_D(x)_6_GSxN signature of NucT is perfectly superimposable with the catalytic centers of Nuc and Zucchini.

### NucT has a strong preference for single-stranded nucleic acids

Some of the structural homologues of NucT carry endonuclease activities, albeit with distinct substrate specificities. The bacterial endonuclease Nuc is active on double- (dsDNA) and on single-stranded DNA (ssDNA), although the relative catalytic efficiencies on those substrates were not reported [2,28,29]. However, the Zucchini endonuclease is highly specific for single-stranded nucleic acids [8]. A preliminary characterization of NucT using high enzyme concentrations (between 1 and 100 μM) had shown that NucT has a strong, thermostable and cation-independent nuclease activity on both RNA and DNA and a preference for ssDNA over dsDNA [10].

To better characterise its enzymatic activities, NucT, deleted of its 23-first amino acids corresponding to the periplasm addressing peptide signal, was purified to near homogeneity (S2 Fig) and its nuclease capacity was tested on different oligonucleotide substrates (Fig 2A). While NucT efficiently degraded ssDNA (substrate I), it had a weaker nuclease activity on dsDNA (substrate II), (Fig 2B). Indeed, the degradation of the full length ssDNA substrate as a function of the amount of added enzyme was at least 3-fold more efficient than that of the dsDNA (Fig 2B). But, because the DNA products released during the first stages of incubation become substrates themselves and therefore compete with the full-length DNA molecule, this 3-fold variation is likely to be an underestimate. To further confirm the preference for ssDNA over dsDNA, the labelled 62-mer was annealed to 32- or 30-mer oligonucleotides complementary to the 5’ or 3’ extremities of the 62-mer to generate substrates III and IV, respectively (Fig 2A). Both the 5’ and 3’ single-stranded overhangs were degraded before the double-stranded region of the substrate, as shown by the persistence of bands corresponding to the 32 and 30 bp double-stranded regions of the substrates (Fig 2C). As expected, NucT had a strong RNase activity, comparable to that on ssDNA substrates (Fig 2D). To rule out a possible contamination of the protein with a RNase, a catalytically inactive mutant was purified in the same conditions (see below and S2B Fig). No nuclease activity was detected on either RNA or ssDNA even at concentrations 10-fold higher than those required for complete degradation of the substrates by the wild-type enzyme (Fig 3A).

To test whether NucT shared the catalytic mechanism with the other characterized nucleases belonging to the PLD family, histidine124 was mutated to an asparagine. The mutant protein behaved as the WT during purification (S2B Fig). NucT^H124N^ was inactive on both ssDNA and RNA substrates (Fig 3A). This result, together with the conserved sequence of the active site, suggests that NucT from *H*. *pylori* shares the catalytic mechanism of Nuc and Zucchini nucleases.

We further used the NucT^H124N^ catalytic mutant to evaluate the affinity of the protein for ss- or dsDNA oligonucleotides using gel retardation assays. NucT bound to a ssDNA 62-mer with an apparent *K*_*D*_ of 2nM (Fig 3B). Interestingly, the *K*_*D*_ for the mouse Zucchini was estimated, using a 50-mer ssDNA or RNA, to be around 50nM [8], implying that NucT has roughly 25-fold higher affinity for its substrate than the mammalian protein. Binding of NucT^H124N^ to ssDNA substrates was also observed for a 32-mer (Fig 3C). However, NucT^H124N^ binding to the dsDNA 62-mer substrate could not be detected (Fig 3B). This is somehow surprising considering the capacity of enzyme to cleave dsDNA. A likely explanation to this apparent contradiction is that the assays were carried out under conditions that, while being standard, do not allow the detection of the binding to dsDNA and therefore exacerbate the difference in affinity for the substrates tested. Taken together, these results indicate that the discrimination between single-stranded and double-stranded substrates results from the different capacities of the protein to bind them.

### NucT is an endonuclease

**Fig 4:**
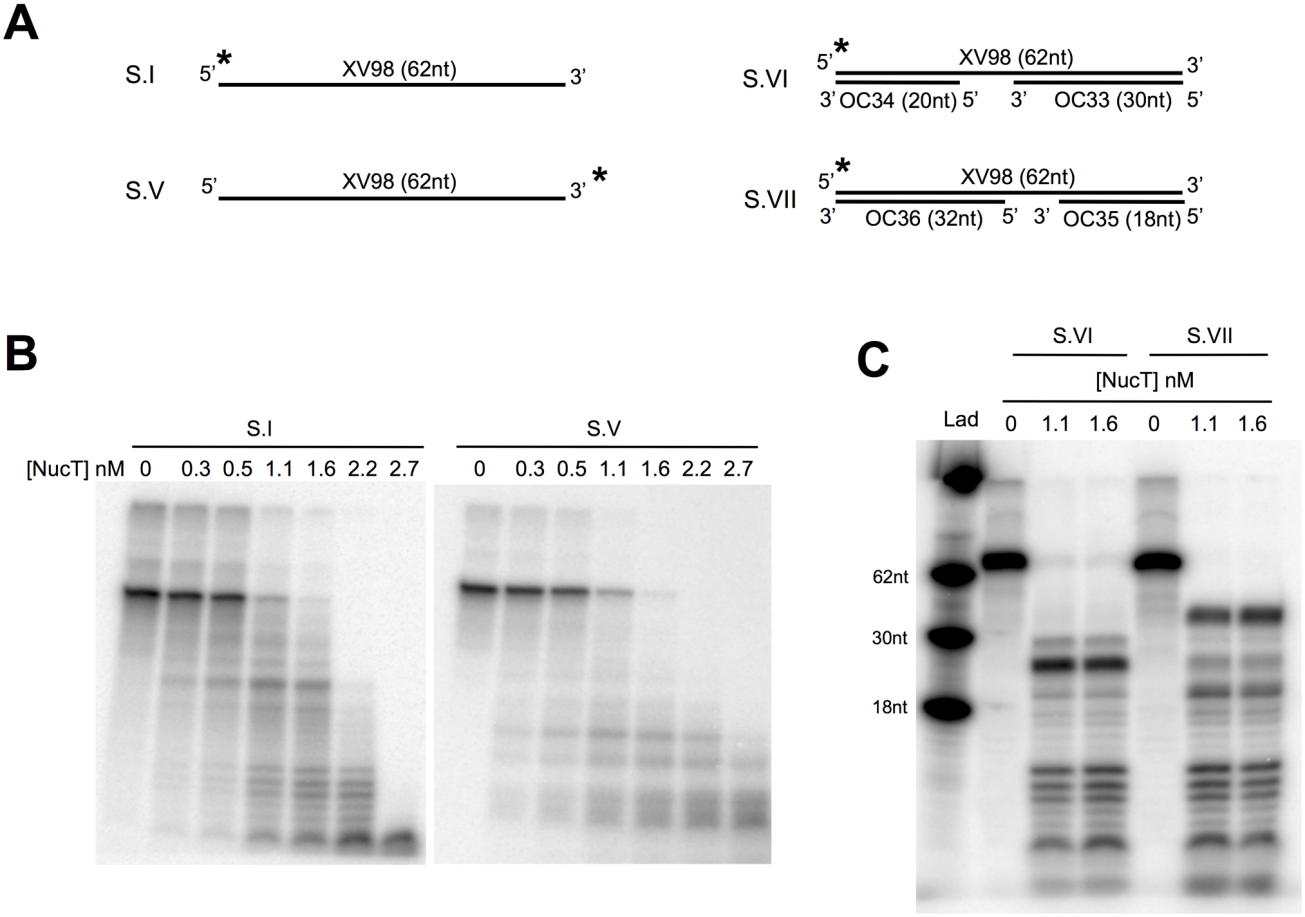
NucT presents endonuclease activity on ssDNA. **(A)** Schematic representation of the different radiolabelled DNA substrates (S.) used. See also the S1 Table. Asterisks indicate the radiolabelled 5’ or 3’ end of the XV98 oligonucleotide. **(B and C)** Nuclease activity tests. Different concentrations of NucT were incubated for 30 min at 37°C with the indicated substrate. The samples were run on denaturing PAGE as described in Materials and Methods. Lad for Ladder: three 5’ radiolabelled different oligonucleotides were used as ladder.

The degradation patterns obtained in the experiment described in the left panel of Fig 2C, showing a discrete double stranded intermediate >30 nucleotides even at very low enzyme concentrations, suggest that NucT acts as an endonuclease. To specifically address this point, the ssDNA 62-mer was labelled with ^32^P at either its 5’ or 3’ ends, to generate substrates I and V, respectively (Fig 4A). If NucT had an exonuclease activity starting on the labelled extremity, the nucleotide carrying the radioactive phosphate should be excised first with the concomitant loss of the band corresponding to the full length substrate without appearance of degradation intermediates. In both cases, there was a gradual loss of the full length substrates accompanied by the appearance of smaller labelled bands (Fig 4B). This result indicates that the nuclease activity is not specific for DNA ends. However, the experiments using double-stranded substrates with extruded single stranded ends (substrates III and IV, Fig 2C) showed that a 3’ single-stranded end makes an about 10-fold better substrate for NucT, suggesting a bias for the loading/binding of the substrate into the active site through a 3’ end.

To definitely show that NucT harbours an endonuclease activity, dsDNA substrates with a single-stranded gap were constructed (substrates VI and VII, Fig 4A). In that case, NucT efficiently degraded the single-stranded region while essentially preserving the double stranded ones, as shown by the appearance of discrete intermediates, thus confirming its endonuclease activity (Fig 4C).

In summary, these results show that NucT has an endonuclease activity with a strong preference for single-stranded DNA (or RNA) substrates, over double-stranded ones. These characteristics are very similar to those of the Zucchini nuclease from mouse [8].

The substrate specificity described above does not provide clear hints about the main function of this protein. The preference for ssDNA, together with its proposed periplasmic localisation, would favour a role in transformation, possibly by degrading the transforming DNA strand complementary to the one entering the cytoplasm. However, the mild transformation phenotype of the *nucT* strains [10,30] indicates that the protein does not have an essential function during competence. On the other hand, It can be assumed that a nuclease activity on dsDNA would be required for its role in purine salvage [12], although the presence of single stranded DNA in the environment cannot be ruled out nor the fact that the activity on dsDNA described could be sufficient for this function.

### The width of the positively charged catalytic groove of NucT explains its preference for single-stranded substrates

**Fig 5:**
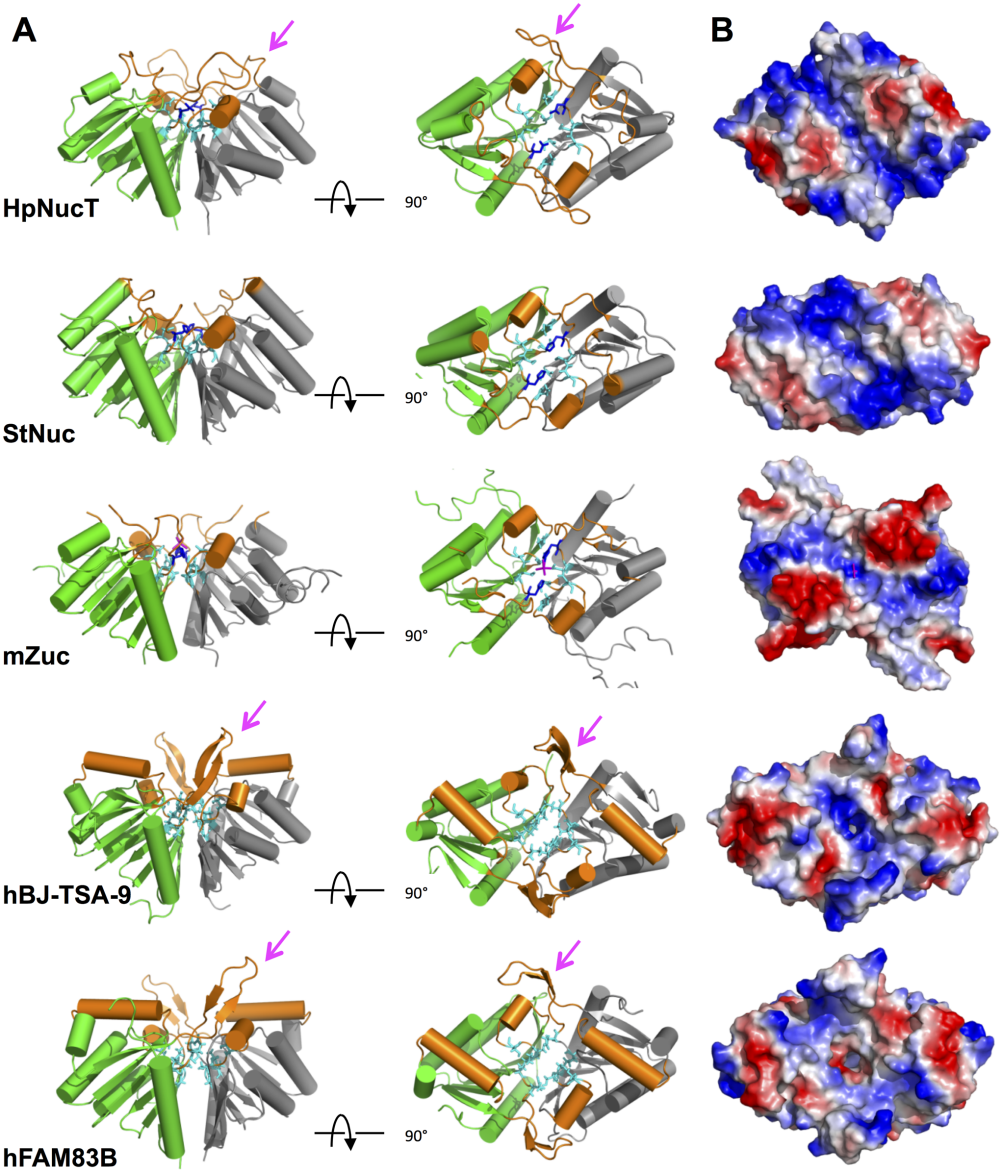
Dimer of NucT and comparative analysis with PLD family nucleases. **(A)** Ribbon diagrams of the dimers of NucT, Nuc, Zuc, hBJ-TSA-9 and hFAM83B in two orientations (profile and on top). One monomer is in green and the other on grey. The helices and loops which line the groove of the catalytic pockets are in orange. The extra loops of NucT, hBJ-TSA-9 and hFAM83B are indicated by a pink arrow. **(B)** Electrostatic surfaces of the top of the dimers. The basic electropositive residues are in blue and the acidic electronegative ones are in red.

NucT formed dimers in the crystal, a feature in common with all the PLD superfamily members described so far. The active site, twinned by the dimerization, is nested in the hollow of its dimer interface, surrounded by two small helices and long loops (orange in Fig 5A). The resulting groove is positively charged (in blue on Fig 5B) but narrow, because flanked by two electronegative patches on both sides (in red in Fig 5B). We have shown in this study that NucT binds the ssDNA but not the dsDNA (Fig 3B). The substrate specificity of NucT (i.e. single-stranded vs double-stranded nucleic acids) could then be determined by the restriction of the accessibility of the active site due to the two negative patches.

A two-step catalytic mechanism involving the two catalytic histidines in the dimer has been proposed for *S*. *typhimurium* Nuc [1,8,31]. The active site of Nuc is also located in the hollow of its dimer interface (Fig 5A). The resulting surface, lined by helices and loops positively charged, is large enough to accommodate a double-stranded B-helix of DNA [1] and is highly positively charged to interact with the polyphosphate DNA backbone (Fig 5B) [2]. In contrast, the Zucchini endonuclease, which is highly specific for single-stranded nucleic acids [8], displays a narrow electropositive surface, flanked by two large electronegative patches, which reduce the accessibility to the active site to smaller substrates, thus adapted for accommodating a single stranded but not a double stranded RNA [8,9] and leading to a lack of activity on double-stranded substrates. The structural characteristics of NucT and Zucchini active sites suggest that these proteins share the same mode of substrate selectivity.

### A basic extra loop for a better binding efficiency?

**Fig 6:**
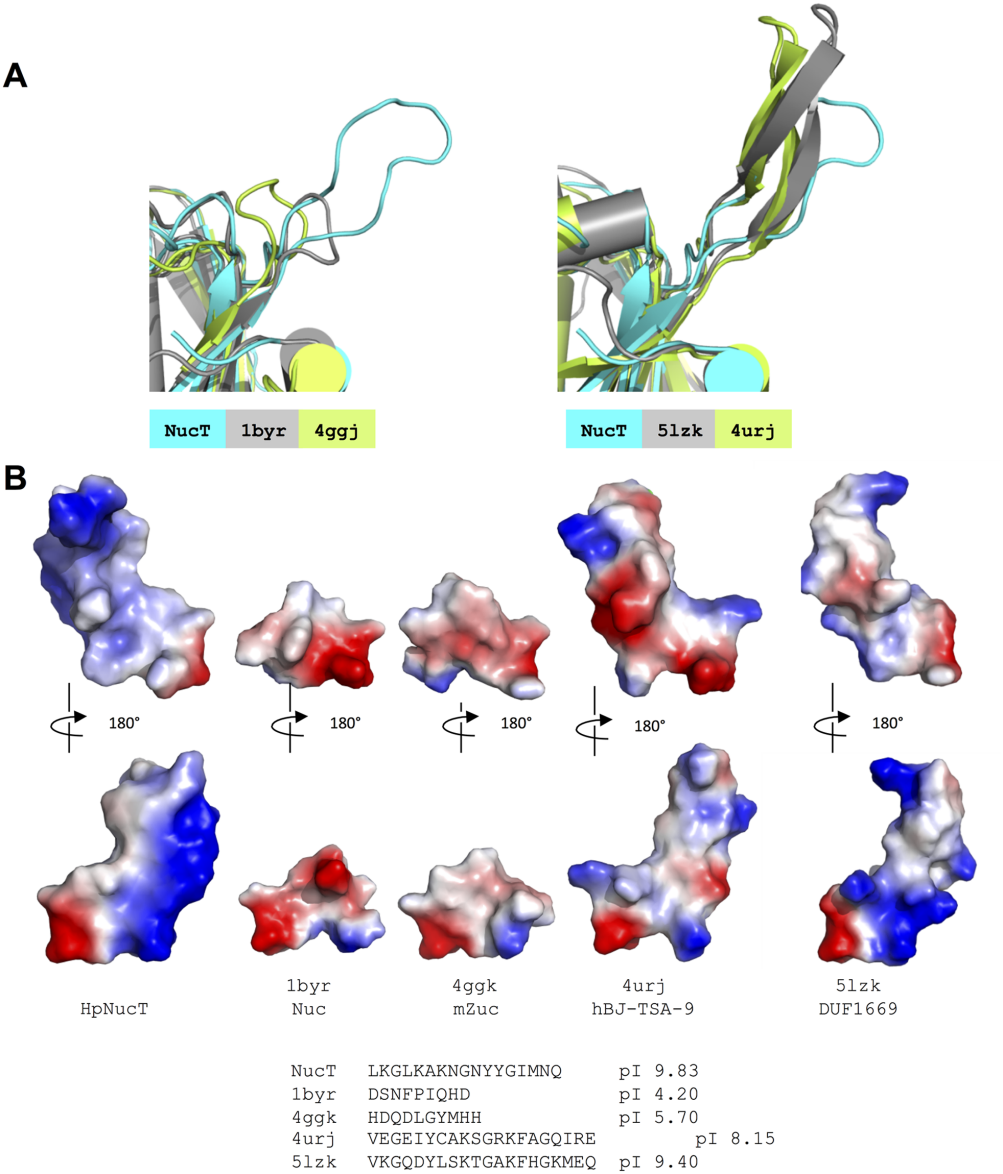
Conformation of the extra loop of NucT. **(A)** Ribbon diagram representation of the superimposition of the extra loop of NucT (cyan) with the short loops of Nuc (grey) and Zucchini (green) (left) and with the extra elements of human BJ-TSA-9 (grey) and human FAM83B (green) (right). **(B)** Electrostatic surfaces of the loops. The basic electropositive residues are in blue and the acidic electronegative ones are in red. The sequences and the isoelectric points of these loops are indicated.

We have shown that NucT shares strong structural similarity with the other members of the nuclease PLD sub-family. However, a major difference between NucT and Nuc or Zucchini resides in the presence of an extra loop present exclusively in the NucT structure (Fig 6A left). This loop participates to enclose the access of the active groove of NucT (Fig 5A). This suggests, within the PLD family, a slight structural variability controlling the access to the active grooves. While the short loops present in Nuc and Zuc are acidic (pI 4.2 and 5.7 respectively), the extra loop in NucT carries a strong positive charge, with a local pI close to 10 (Fig 6B). Thus, the NucT loop might contribute to attract the DNA into the electrostatic groove of the dimer, resulting in the higher affinity for the ssDNA when compared to Zuc. The strong positive charge of the loop might also explain the capacity, absent in Zuc, to act on dsDNA, albeit with less efficiency. Interestingly, alignment of the NucT sequences from Helicobacteraceae indicates that this loop constitutes a variable region (S1 Fig), suggesting that the enzyme might present different affinities for its substrates within the family. While the sequence variability within the Helicobacteraceae family makes it unlikely, other roles for this loop such as interacting with periplasmic or membrane proteins, cannot be ruled out.

Two other human proteins share considerable structural similarity with NucT: 5lzk (human FAM83B, DUF1669) and 4urj (human BJ-TSA-9), with rmsd values after superposition with NucT of 1.68 and 1.95 Å, respectively (Fig 5). The functions of these two proteins are unknown. Interestingly, the consensus sequences (ExK(x)4D(x)6GSxS and RxK(x)4D(x)6GSxS, respectively), lack the catalytic histidine of the Phospholipase D superfamily and these proteins may therefore have different catalytic (or non-catalytic) functions. The extra loop of NucT which has no regular secondary structure is replaced in the human structural analogs by a connection composed of two short β-strands composed of basic residues (Fig 6A right). They all occupy the same steric hindrance and are all globally basic (8.1 and 9.4 respectively for BJ-TSA-9 and FAM83B and near 10 for NucT) (Fig 6B). These characteristics suggest that these extra loops share the same role in the proteins although their functions remain unknown.

In sum, the combination of the crystal structure determination and the substrate specificity characterization for NucT and their comparison with those of other members of the PLD family allowed us to propose a model by which the specificity of the enzyme for single-stranded substrates is defined by the width of the acidic groove into which the catalytic site is found.

## Accession number

The atomic coordinates and structure factors of NucT have been deposited at the Brookhaven Protein Data Bank under the accession number 6EHI.

## Supporting information

S1 Fig Sequence alignment of the NucT proteins. Sequence alignment of the bacterial NucT proteins.
(TIFF)

S2 Fig Purification of NucT. SDS–polyacrylamide gel illustrating the overproduction and purification of NucT^H124N^. M, size molecular marker (Precision Plus Protein Unstained Standart from BioRad); NI and Ind, whole-cell extracts of uninduced and induced cultures respectively; C.O.E, Cold Osmotic Extract; Amylose, purified MBP-NucT^H124N^ obtained after elution on amylose resin column; TEV, precedent fraction after digestion by TEV protease; Res.S, purified NucT^H124N^ obtained after elution of TEV digested fraction on a Resource S column. The arrow indicates the position of the different proteins. For details see Materials and methods.
(TIF)

S1 Table Oligonucleotides used for the activity tests. List and sequences of oligonucleotides used as substrates for NucT activity and binding assays.
(DOCX)
