# Supplementary figures and images for: Structural basis for the substrate selectivity of Helicobacter pylori NucT nuclease activity
Source: PLoS One. 2017 Dec 4;12(12):e0189049. doi: 10.1371/journal.pone.0189049 (PMC5714352; doi:10.1371/journal.pone.0189049)

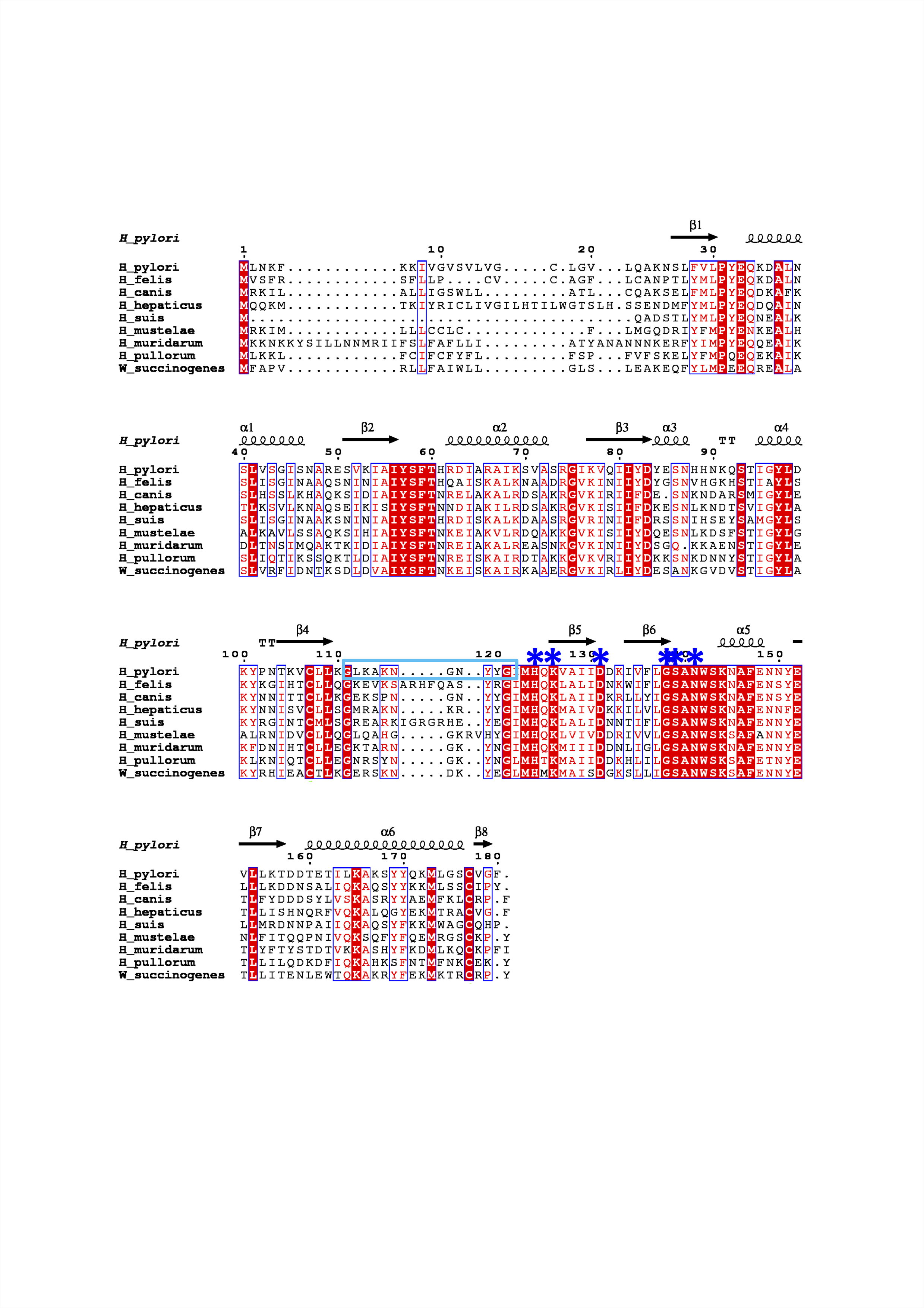

Supplement: S1 Fig — Sequence alignment of the bacterial NucT proteins. (TIFF) [file pone.0189049.s001.tiff]

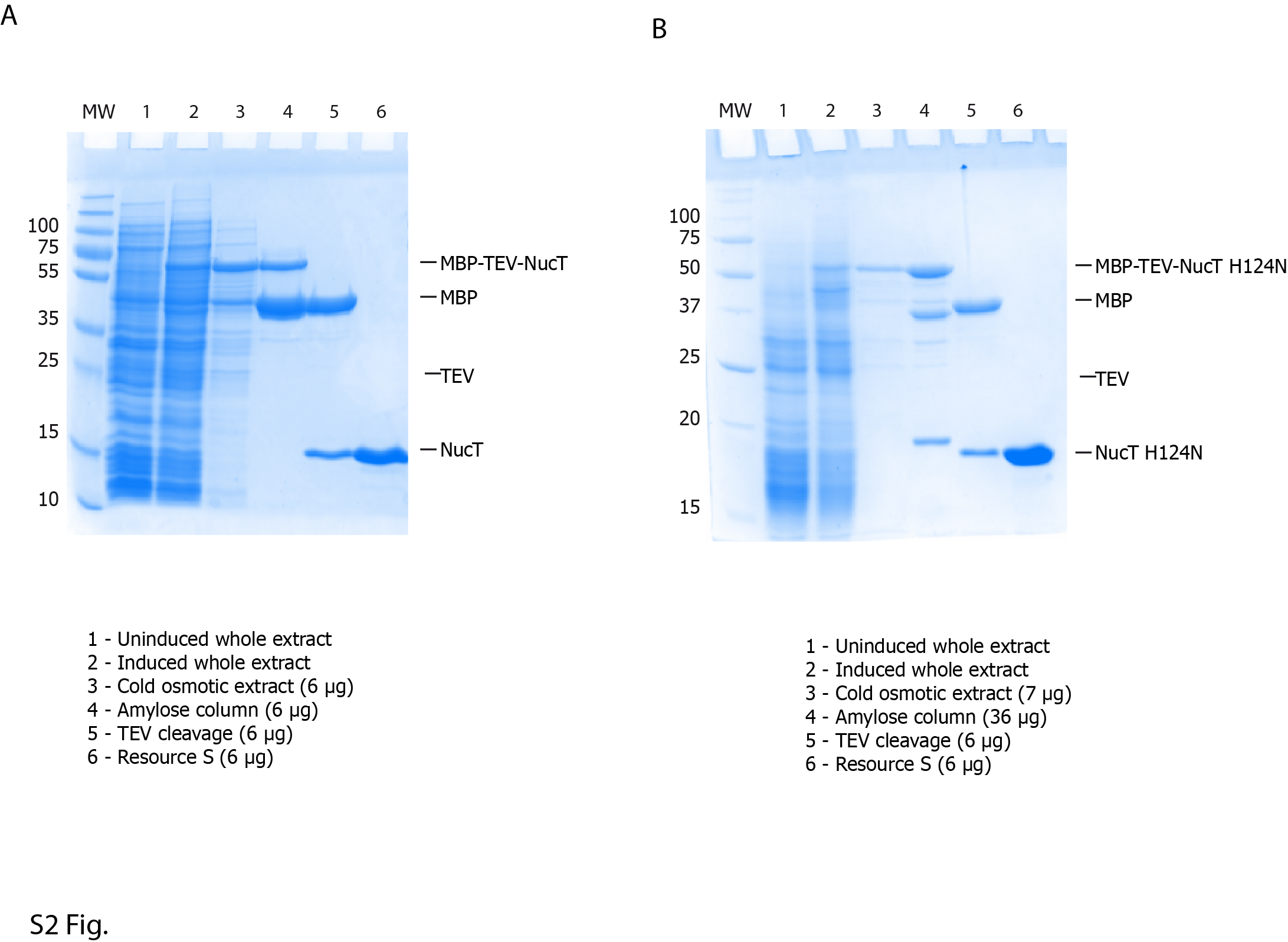

Supplement: S2 Fig — SDS–polyacrylamide gel illustrating the overproduction and purification of NucTH124N. M, size molecular marker (Precision Plus Protein Unstained Standart from BioRad); NI and Ind, whole-cell extracts of uninduced and induced cultures respectively; C.O.E, Cold Osmotic Extract; Amylose, purified MBP-NucTH124N obtained after elution on amylose resin column; TEV, precedent fraction after digestion by TEV protease; Res.S, purified NucTH124N obtained after elution of TEV digested fraction on a Resource S column. The arrow indicates the position of the different proteins. For details see Materials and methods. (TIF) [file pone.0189049.s002.tif]
