# Supplementary material for: Structural basis for the substrate selectivity of Helicobacter pylori NucT nuclease activity
Source: PLoS One. 2017 Dec 4;12(12):e0189049. doi: 10.1371/journal.pone.0189049 (PMC5714352; doi:10.1371/journal.pone.0189049)
Supplement: S1 Table — List and sequences of oligonucleotides used as substrates for NucT activity and binding assays. (DOCX) [file pone.0189049.s003.docx]

**S1 Table: Oligonucleotides used for the activity tests**

| name | size | sequence |
| --- | --- | --- |
| XV98 | 62 | 5’- CAACGGCATAAAGCTTGACGATTACATTGCTAGGACATCTTTGCCCACCTGCAGGTTCACCC -3’ |
| OC32 | 62 | 5’-GGGTGAACCTGCAGGTGGGCAAAGATGTCCTAGCAATGTAATCGTCAAGCTTTATGCCGTTG-3’ |
| OC34 | 20 | 5’-CGTCAAGCTTTATGCCGTTG-3’ |
| OC33 | 30 | 5’-GGGTGAACCTGCAGGTGGGCAAAGATGTCC-3’ |
| OC36 | 32 | 5’-TAGCAATGTAATCGTCAAGCTTTATGCCGTTG-3’ |
| OC35 | 18 | 5’-GGGTGAACCTGCAGGTGG-3’ |
| osf344 | 24 | 5’-CGCAGCTCACTTTAAGGACTTTCC-3’ |
| Hxv82 | 25 | 5’-CCAAUGUCCUUGACGCUGUCUAGAG-3’ |
